# Effects of bergapten on the pharmacokinetics of macitentan in rats both *in vitro* and *in vivo*

**DOI:** 10.3389/fphar.2023.1204649

**Published:** 2023-07-10

**Authors:** Jia Xu, Quan Zhou, Pengjiao Hou, Yu Wang, Peiwu Geng, Zebei Lu, Yunfang Zhou, Dapeng Dai, Shuanghu Wang

**Affiliations:** ^1^ Department of Pharmacy, The Sencond Affiliated Hospital of Jiaxing University, Jiaxing, Zhejiang, China; ^2^ The Laboratory of Clinical Pharmacy, The Sixth Affiliated Hospital of Wenzhou Medical University, Lishui People’s Hospital, Lishui, Zhejiang, China; ^3^ The Key Laboratory of Geriatrics, Beijing Institute of Geriatrics, Institute of Geriatric Medicine, Chinese Academy of Medical Sciences, Beijing Hospital/National Center of Gerontology of National Health Commission, Beijing, China

**Keywords:** pulmonary arterial hypertension, macitentan, bergapten, drug-drug interaction, pharmacokinetics

## Abstract

Macitentan was approved by the United States Food and Drug Administration (FDA) in 2013 for the treatment of pulmonary arterial hypertension (PAH). Bergapten is a furanocoumarin that is abundant in Umbelliferae and Rutaceae plants and is widely used in many Chinese medicine prescriptions. Considering the possible combination of these two compounds, this study is aimed to investigate the effects of bergapten on the pharmacokinetics of macitentan both *in vitro* and *in vivo*. Rat liver microsomes (RLMs), human liver microsomes (HLMs), and recombinant human CYP3A4 (rCYP3A4) were used to investigate the inhibitory effects and mechanisms of bergapten on macitentan *in vitro*. In addition, pharmacokinetic parameters were also studied *in vivo*. Rats were randomly divided into two groups (six rats per group), with or without bergapten (10 mg/kg), and pretreated for 7 days. An oral dose of 20 mg/kg macitentan was administered to each group 30 min after bergapten or 0.5% CMC-Na administration on day 7. Blood was collected from the tail veins, and the plasma concentrations of macitentan and its metabolites were assessed by ultra-performance liquid chromatography - tandem mass spectrometer (UPLC-MS/MS). Finally, we analyzed the binding force of the enzyme and two small ligands by *in silico* molecular docking to verify the inhibitory effects of bergapten on macitentan. The *in vitro* results revealed that the IC_50_ values for RLMs, HLMs, and rCYP3A4 were 3.84, 17.82 and 12.81 μM, respectively. *In vivo* pharmacokinetic experiments showed that the AUC_(0-t)_, AUC_(0-∞)_, and C_max_ of macitentan in the experimental group (20,263.67 μg/L*h, 20,378.31 μg/L*h and 2,999.69 μg/L, respectively) increased significantly compared with the control group (7,873.97 μg/L*h, 7,897.83 μg/L*h and 1,339.44 μg/L, respectively), while the CL_z_/F (1.07 L/h/kg) of macitentan and the metabolite-parent ratio (MR) displayed a significant decrease. Bergapten competitively inhibited macitentan metabolism *in vitro* and altered its pharmacokinetic characteristics *in vivo*. Further molecular docking analysis was also consistent with the experimental results. This study provides a reference for the combined use of bergapten and macitentan in clinical practice.

## 1 Introduction

Pulmonary arterial hypertension (PAH) is a progressive and fatal pulmonary vascular disease that results from an imbalance in vasoactive substances released by the pulmonary vascular endothelium. It is characterized by an increase in pulmonary vascular resistance, ultimately leading to right-sided heart failure and death (Galiè et al, 2013). It is estimated that 15–50 people per million in a population are affected by PAH. It is the first of the five general categories of pulmonary hypertension that can be idiopathic, inherited, or consequences of drugs, toxins, or other disorders (Peacock et al, 2007; Degano et al, 2009; Hoeper et al, 2013; Ahmed and Palevsky, 2014; Gashouta et al, 2014). To date, improving symptoms, slowing disease progression, and increasing the survival rate are the main aims of PAH treatment (Galiè et al, 2013). To regulate the pulmonary vascular pressure, prostacyclin, nitric oxide, and endothelin (ET) pathways play important roles in clinical treatment (Chester and Yacoub, 2014). ET-1 is a potent vasoactive peptide that promotes endothelial dysfunction and vascular remodeling, eliciting its effects through mediating vascular contraction (ETA) and vascular dilatation (ETB) receptors (Brunner et al, 2006). The balance maintained by the regulation of ETA and ETB ensures a normal pulmonary vascular environment.

**FIGURE 1:**
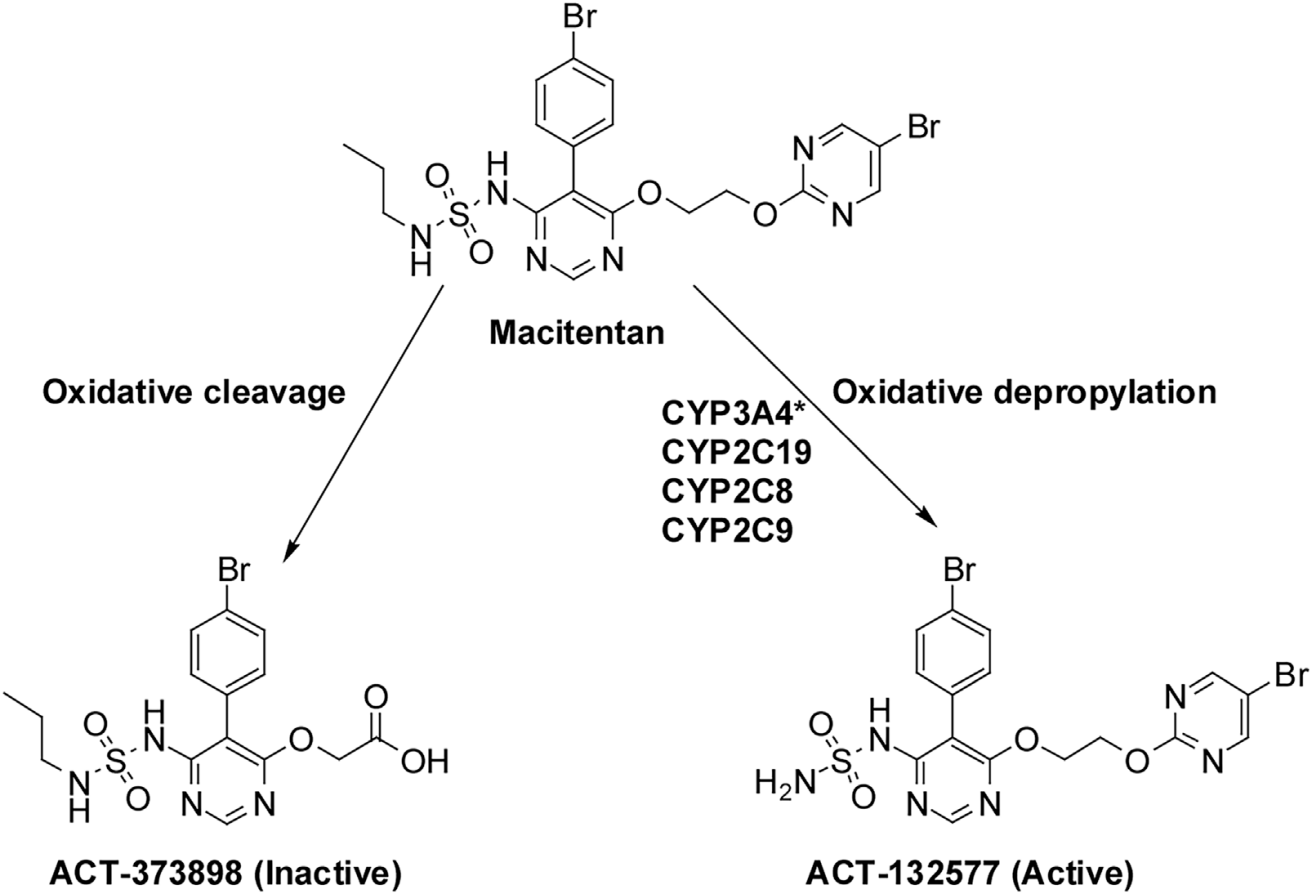
Macitentan metabolism to ACT-132577 and ACT-373898. Notes: * Main metabolic pathway.

The selective antagonism of ETA receptors is undoubtedly the first choice for the treatment of PAH, owing to the vasoconstriction function of ETA. However, the renin-angiotensin system is activated by the specific blockade of ETA receptors, resulting in edema (Schirger et al, 2004; Patel et al, 2015). Therefore, this adverse event can be avoided by dual antagonism of both ETA and ETB receptors, making the treatment more effective and safer (Sauvageau et al, 2007). Macitentan is a second-generation dual endothelin receptor antagonist approved in 2013 (Patel and McKeage, 2014). Compared with other endothelin receptor antagonists, macitentan exhibits the advantages of optimized receptor occupancy, enhanced tissue penetration, and improved liver safety profile (Treiber et al, 2014). ACT-132577 and ACT-373898 are the two main metabolites of macitentan formed by oxidative depropylation and oxidative cleavage, respectively (Bruderer et al, 2012a). ACT-132577 is pharmacologically active and can contribute to the antagonism of the ET receptor, similar to macitentan, although its efficacy is approximately five-fold less potent than macitentan (Iglarz et al, 2008). To our knowledge, the cytochrome P450 (CYP) system plays an important role in the metabolism and overall elimination of drugs. It has been shown that the formation of ACT-132577 is mainly catalyzed by CYP3A4, with minor contributions from CYP2C8, CYP2C9, and CYP2C19 (Ohnishi et al, 2000; Patel and McKeage, 2014) (Figure 1). Induction or inhibition of CYP enzymes usually leads to changes in the pharmacokinetics and pharmacodynamics of specific drugs. CYP3A is the largest subfamily of CYP enzymes expressed in the human liver and gastrointestinal tract and shows great potential in the metabolism of most clinical drugs (Hu et al, 2017). Accordingly, based on the metabolism by CYP3A4, much attention should be paid to the drug-drug interactions (DDIs) between macitentan and other CYP3A4 inducers and inhibitors.

Anticoagulant therapy is one of the main treatments for patients with PAH, especially for patients with primary pulmonary hypertension and chronic thromboembolic pulmonary hypertension (Alpert et al, 1994; Johnson et al, 2006; Porres-Aguilar et al, 2021). Owing to the basic structure of benzo-[alpha]-pyrone, coumarins can compete with vitamin K and affect the activation of coagulation factors, thus exhibits the anticoagulant effect in clinic. Warfarin is one of the most commonly used oral coumarin anticoagulants, which play an important role in the anticoagulation treatment of PAH (Opitz et al, 2009). Recently, DDIs between warfarin and macitentan have been well studied in healthy male subjects, which encourage us to explore whether some potential DDIs are existed between other coumarins and PAH treatment drug macitentan (Sidharta et al, 2014).

Bergapten (BP), also known as 5-methoxypsoralen, is a natural furocoumarin which could be widely found in Umbelliferae plants, such as *Cnidii Fructus* (Chen et al, 2009), *Angelica dahurica* (Ban et al, 2003), and *Peucedanum ostruthium* (Vogl et al, 2011). With the basic structure of benzo-[alpha]-pyrone, furocoumarins display various pharmacological activities, including anti-inflammatory (Tuan Anh et al, 2017), analgesic effects (Singh et al, 2019), and effects on cardiovascular diseases (Liu et al, 2017). It has been reported that BP could exhibit better anticoagulant effect than warfarin in some conditions. For example, the furocoumarins extract containing 35.18% bergapten could promote the normalization of hemostatic potential in the correction of hemostasis distribution for chemotherapy drug cisplatin treated mice model. In contrast, warfarin commonly can provoke the hypocoagulation in that model (Mira et al, 2017). In another mice model for the evaluation of antiplatelet and anticoagulant activities of *Angelica shikokiana* extract, bergapten was found to be one of the main coumarins against adenosine 5′-diphosphate-induced platelet aggregation (Filonova et al, 2021). To date, no studies have been reported on the potential DDIs between the furocoumarin bergapten and PAH treatment drug macitentan.

The present study aimed to comprehensively investigate the effects of BP on the metabolism and pharmacokinetics of macitentan *in vitro* and *in vivo*. Rat liver microsomes (RLMs), human liver microsomes (HLMs), and recombinant human CYP3A4 (rCYP3A4) incubation systems were used to determine the inhibitory effects and mechanisms. Subsequently, an *in vivo* pharmacokinetic investigation was conducted in rats. Quantification of all compounds involved in this study were conducted with an UPLC-MS/MS method, which was extensively used for determination of various analytes owing to its advantages of sensitive, fast and accurate (Amer et al, 2017; Attwa et al, 2018; Al-Shakliah et al, 2020; AlRabiah et al, 2020; Attwa et al, 2020). Finally, a molecular docking study was used to understand the pattern of interaction between two small ligands and CYP3A4 to verify and explain the results obtained *in vitro* and *in vivo*.

## 2 Materials and methods

### 2.1 Chemicals and reagents

BP (Cat No. A0426, purity>98%) was obtained from Chengdu Must Bio-Technology Co., Ltd (Chengdu, China), macitentan and its metabolite ACT-132577 (Cat No. T7817, purity>98%) were purchased from TargetMol Chemicals Inc (Boston, United States), and diazepam (Cat No. 33210701, purity>98%) (used as an internal standard) was obtained from Shandong Viwit Pharmaceutical Co., Ltd., (Shandong, China). Formic acid (Cat No. F112034, purity>98%) was obtained from aladdin (Shanghai, China). Methanol (Cat No. 1.06007, purity>99.8%) and acetonitrile (Cat No. 1.00030, purity>99.8%) were purchased from Merck (Billerica, MA, United States). Ultra-pure water was obtained using a Milli-Q Plus filtration system (Millipore, Billerica, MA, United States). Reduced NADPH (Cat No. C804424, purity>98%) was purchased from Macklin (Shanghai, China). RLMs were prepared in our laboratory (Chen et al, 2020; Zhou et al, 2020). HLMs were purchased from PrimeTox (Wuhan, China). rCYP3A4 was prepared according to the method we previously described (Fang et al, 2017). All other reagents used were of the highest commercially available grade.

### 2.2 Animals

Sprague-Dawley rats (male, 180–220 g) were obtained from the Experimental Animal Center of the Wenzhou Medical University. Animal experiments were approved by the Animal Care and Use Committee of Wenzhou Medical University (approval no. wydw 2019–650). Rats were acclimatized to a light/dark cycle of 12/12 h for at least 1 week. They had free access to water and food under a relative humidity of 40%–60% at 20°C–26°C. The research was conducted in accordance with internationally accepted principles for the care and use of laboratory animals.

### 2.3 Equipment and operation conditions

The concentrations of macitentan and ACT-132577 were determined by UPLC-MS/MS, which consisted of an Acquity I-class UPLC system (Waters Corp., Milford, MA, United States) and a Xevo TQD triple quadrupole mass spectrometer (Waters Corporation, Milford, MA, United States) equipped with an electrospray ionization source. Chromatographic separation was performed on an Acquity UPLC BEH C18 column (2.1 × 100 mm, 1.7 μm) at 40°C. The mobile phase was composed of acetonitrile (solvent A) and water containing 0.1% formic acid (solvent B, pH 2.74). The gradient condition of mobile phases was set with a flow rate of 0.4 mL/min as follows: 0–0.5 min, 20% A; 0.5–1 min, 70% A; 1–2 min, 95% A; 2–2.6 min, 20% A. Positive ion and selective multiple reaction monitoring (SRM) modes were used for quantitative analysis with the precursor to product ion transitions of m/z 588.9→203.4 for macitentan, 546.9→200.6 for ACT-132577, and 285.0→193.0 for diazepam. The cone voltage and collision voltage were 28V, 14 V for macitentan, 26V, 28 V for ACT-132577, and 35V, 30 V for diazepam. The mass spectrometry parameters of the source temperature, desolvation temperature, cone gas flow rate, and desolvation gas flow rate were 150°C, 500°C, 50 L/h, and 1000 L/h, respectively.

### 2.4 Method validation

Method validation, including accuracy, precision, recovery, matrix effects and stability, was conducted in accordance with the guidelines of the FDA and the European Medicines Agency.

### 2.5 Inhibitory effects of BP on macitentan in RLMs, HLMs, and rCYP3A4

The effects of BP on macitetan metabolism *in vitro* were investigated using RLMs, HLMs, and rCYP3A4. And, the formation of ACT-132577 was considered as an indicator of inhibitory effect. The 200 μL incubation system contained NADPH (1 mM), potassium phosphate buffer (100mM, pH = 7.4), macitentan, BP, and RLMs (0.3 mg/mL), HLMs (0.2 mg/mL), or rCYP3A4 (0.13 mg/mL). Firstly, a series concentration of macitentan with 1, 2.5, 5, 10, 25, 50 and 100 μM were included in the reaction buffer to determine the K_m_ and V_max_ values. Then, a series concentration of BP following 0, 0.01, 0.1, 1, 5, 10, 50 and 100 μM were used for the determination of IC_50_ value of BP against macitentan. Finally, to determine the inhibitory mechanism of BP on macitentan, a series concentration of BP was generated in accordance with the IC_50_ levels as follows: 0, 1, 2, 4, and 8 μM for RLMs; 0, 4.5, 9, 18, and 36 μM for HLMs; and 0, 3, 6, 12, and 24 μM for rCYP3A4. The concentrations of macitentan were set in accordance with the K_m_ values, as follows:1.25, 2.5, 5, and 10 μM for RLMs; 1, 2, 4, and 8 μM for HLMs; and 2.5, 5, 10, and 20 μM for rCYP3A4. The system was pre-incubated at 37°C for 5 min, and 10 μL NADPH was added to initiate the reaction. After incubation for 30 min at 37°C, 200 μL ice-cold acetonitrile and 30 μL diazepam (500 ng/mL) were added to terminate the reaction. The samples were centrifuged at 13,000 rpm for 5 min, and the supernatant fraction (150 μL) was collected for UPLC-MS/MS analysis.

### 2.6 Pharmacokinetic interactions between BP and macitentan in rats

Twelve Sprague-Dawley rats were randomly assigned to two groups. BP (10 mg/kg) was continuously administered daily to rats in the experimental group for 7 days, whereas the 0.5% CMC-Na was orally administered once a day to rats in the control group. Rats were fasted overnight before dosing, with free access to water. Macitentan (20 mg/kg) was intragastrically administered 30 min after the last dose of BP or 0.5% CMC-Na on the seventh day. Blood samples (300 μL) were collected from the tail vein into 1.5 mL Eppendorf tubes containing 10 μL heparin sodium at the time points of 0.083, 0.25, 0.5, 1, 2, 3, 4, 6, 8, 12, 24, and 36 h after macitentan administration. Plasma was obtained after centrifugation of the blood samples at 4,000 rpm for 10 min and stored at −80°C for further analysis. Before UPLC-MS/MS analysis, 100 μL plasma was mixed with 200 μL acetonitrile and 30 μL diazepam (500 ng/mL) and then fractionated at 13,000 rpm for 5 min at 4°C. A 150 μL sample of the supernatant was collected for analysis.

### 2.7 Molecular docking simulations

The protein crystal structure was extracted from the Protein Data Bank (PDB ID:2J0D; https://www.rcsb.org/). The 3D structures of BP and macitentan were downloaded from the PubChem platform as sdf format files and converted to PDB files using OpenBabel3.1.1. Finally, the 3D protein conformations were optimized by preprocessing the protein crystal water and adding hydrogen atoms using AutoDockTools 1.5.7. Molecular docking was performed using AutoDock Vina (version 1.1.2). A size of 60 Å × 60 Å × 60 Å was set for the grid box, and the grid spacing was set to 0.3753 Å.

### 2.8 Statistical analysis

All assays were performed in triplicate, and the results are expressed as mean ± standard deviation. The pharmacokinetic parameters of elimination half-life (t_1/2_), area under the plasma concentration-time curve (AUC), maximal plasma concentration (C_max_), time to peak plasma concentration, plasma clearance (CL_z_/F), apparent volume of distribution, and mean residence time were calculated using a non-compartment model with DAS software (version 3.2.8, Lishui People’s Hospital, China). Data analysis, statistical comparisons, and graphs were generated using GraphPad Prism software (version 7.0; GraphPad Software Inc., San Diego, CA, United States). Student’s t-test of variance following the least significant difference method was used for comparisons between the control and experimental groups. Statistical significance was set at *p* < 0.05.

## 3 Results

### 3.1 Method validation

The UPLC-MS/MS method of macitentan and ACT-132577 met the requirements of the bioanalytical methods of FDA and European Medicines Agency, and detailed precision, recovery, matrix effects and stability evaluation results were shown in Table 1 and Table 2, respectively. The linear ranges for the analytes are as following: 2, 5, 10, 20, 50, 100,200, 500, 1,000, 2,000 ng/mL for macitentan, 10, 25, 50,100, 250, 500, 1,000, 2,500, 5,000, 10,000 ng/mL for ACT-132577.

**TABLE 1:** Evaluation of the Intra- and Inter-day precision, recovery and matrix effects by the proposed UPLC-MS/MS method for determination of macitentan and ACT-132577 in rat plasma (*n* = 6).

Analytes	Preparation concentration (ng/mL	Intra-day		Inter-day		Recovery (%)	Matrix effects (%)
		Precision RSD (%)	Accuracy RE (%)	Precision RSD (%)	Accuracy RE (%)		
Macitentan	4	6.90	10.05	9.36	8.62	86.43	86.16
	160	5.24	6.57	2.79	7.59	95.94	92.87
	1,600	4.79	9.55	3.67	7.77	90.91	91.54
ACT-132577	30	7.01	11.52	6.83	8.74	88.12	89.77
	900	4.53	8.49	5.9	5.83	90.40	94.02
	9000	3.68	2.28	2.49	2.92	92.18	93.18

Abbreviations: RSD, relative standard division; RE, relative error.

**TABLE 2:** Stability evaluation results of macitentan and ACT132577 in rat plasma under different conditions (*n* = 6).

Analytes	Preparation concentration (ng/mL)	Room temperature, 6 h		4°C, 24 h		−80°C, 7days	
		RSD (%)	RE (%)	RSD (%)	RE (%)	RSD (%)	RE (%)
Macitentan	4	4.72	113.51	4.44	107.57	5.33	104.39
	160	7.94	109.43	2.28	104.86	2.34	103.80
	1,600	3.30	105.63	3.47	106.50	2.92	105.44
ACT-132577	30	7.56	109.84	8.71	106.81	6.58	105.76
	900	8.27	106.18	5.60	104.77	3.20	107.26
	9000	8.50	107.44	3.35	105.28	4.67	103.40

Abbreviations: RSD, relative standard division; RE, relative error.

### 3.2 Evaluation of the influence of BP on macitentan metabolism *in vitro*

**FIGURE 2:**
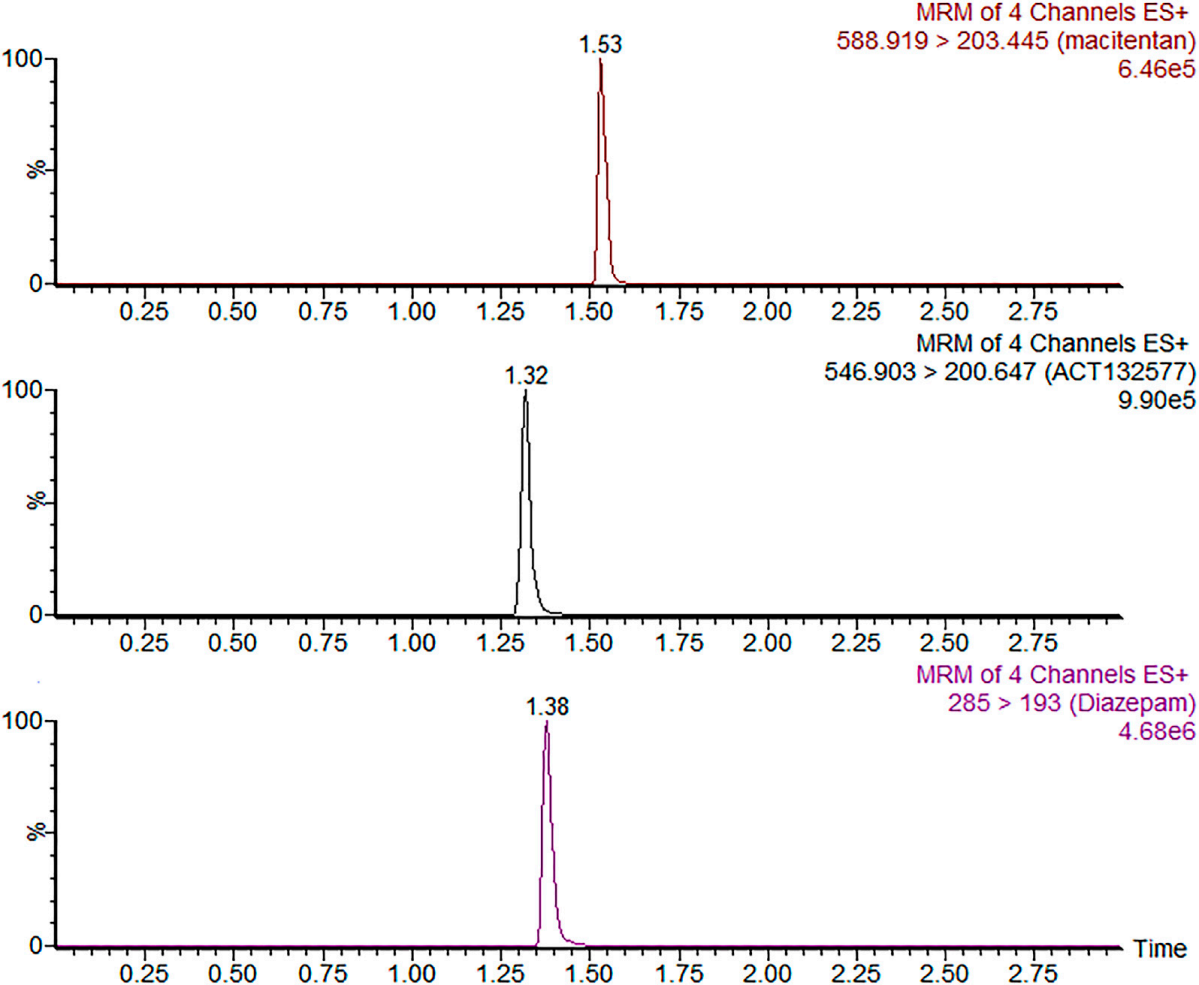
SRM chromatograms of macitentan, ACT-132577 and IS.

**FIGURE 3:**
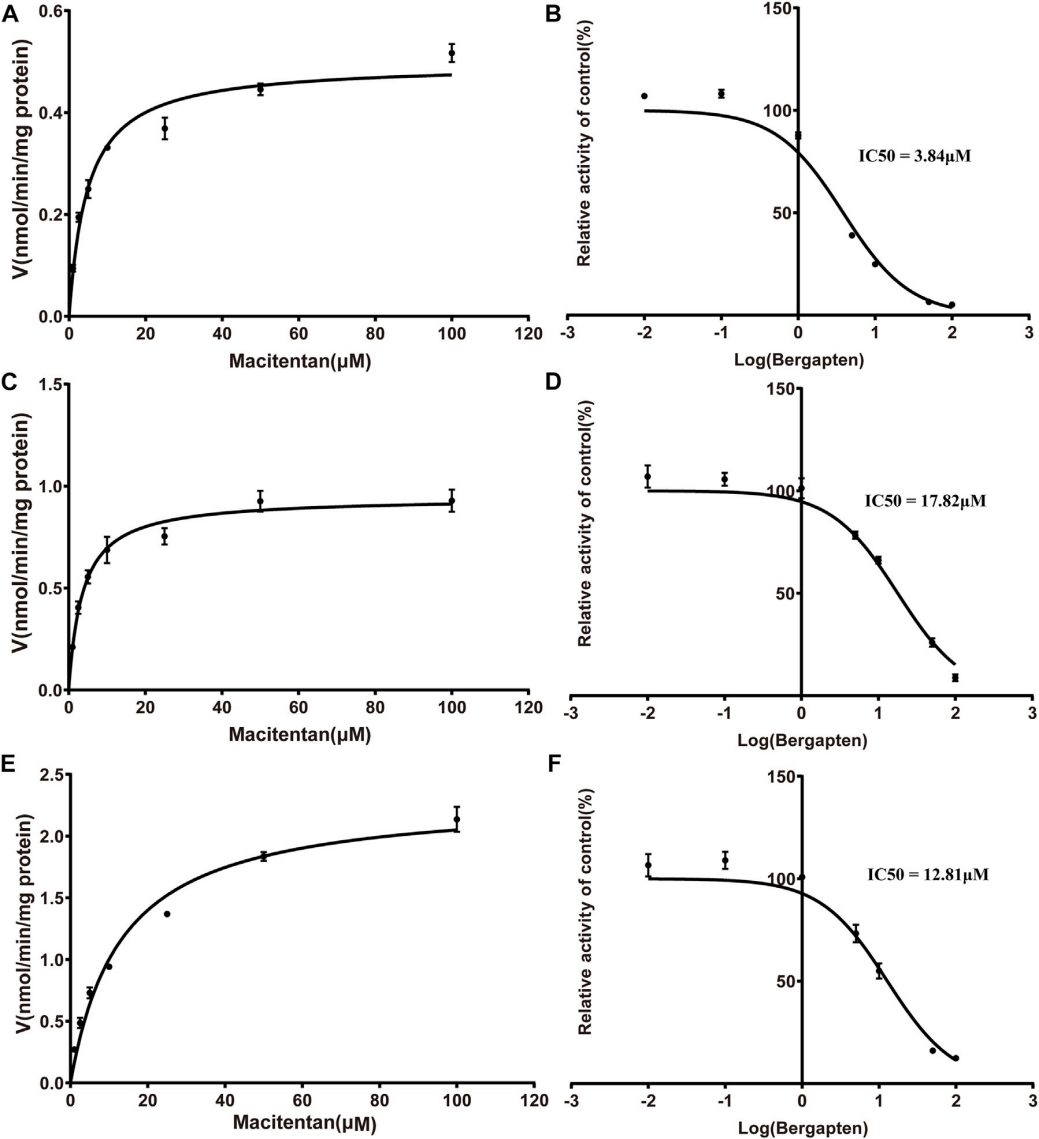
Michaelis-Menten kinetics and IC_50_ plots of effect of bergapten on macitentan in RLMs **(A, B)**, HLMs **(C, D)** and human recombinant **(E, F)** (*n* = 3, mean ± SD).

**FIGURE 4:**
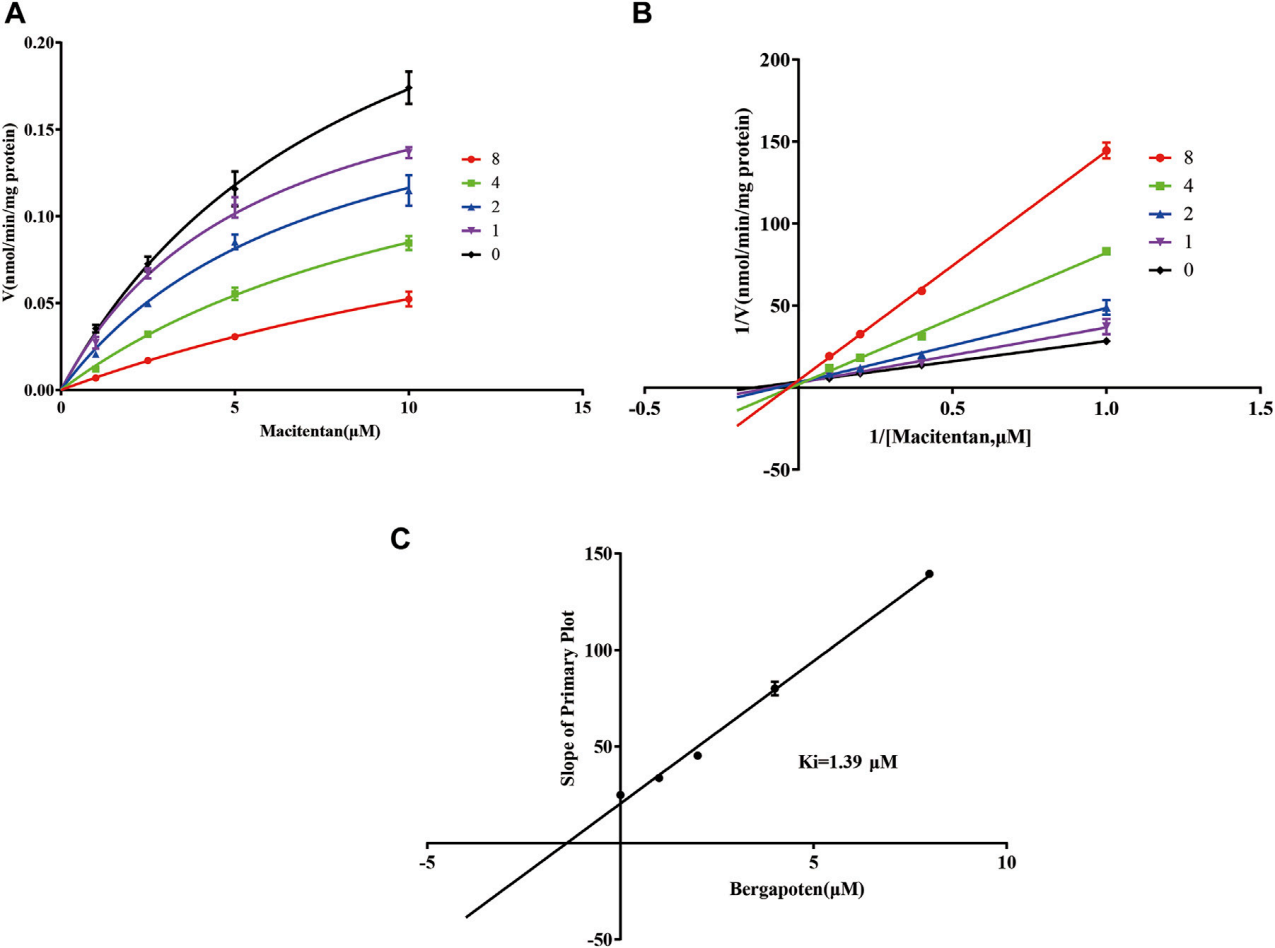
Michaelis-Menten model **(A)**, Lineweaver–Burk plots **(B)**, and slope of primary plot **(C)** for bergapten inhibition of macitentan in RLMs (*n* = 3, mean ± SD).

**FIGURE 5:**
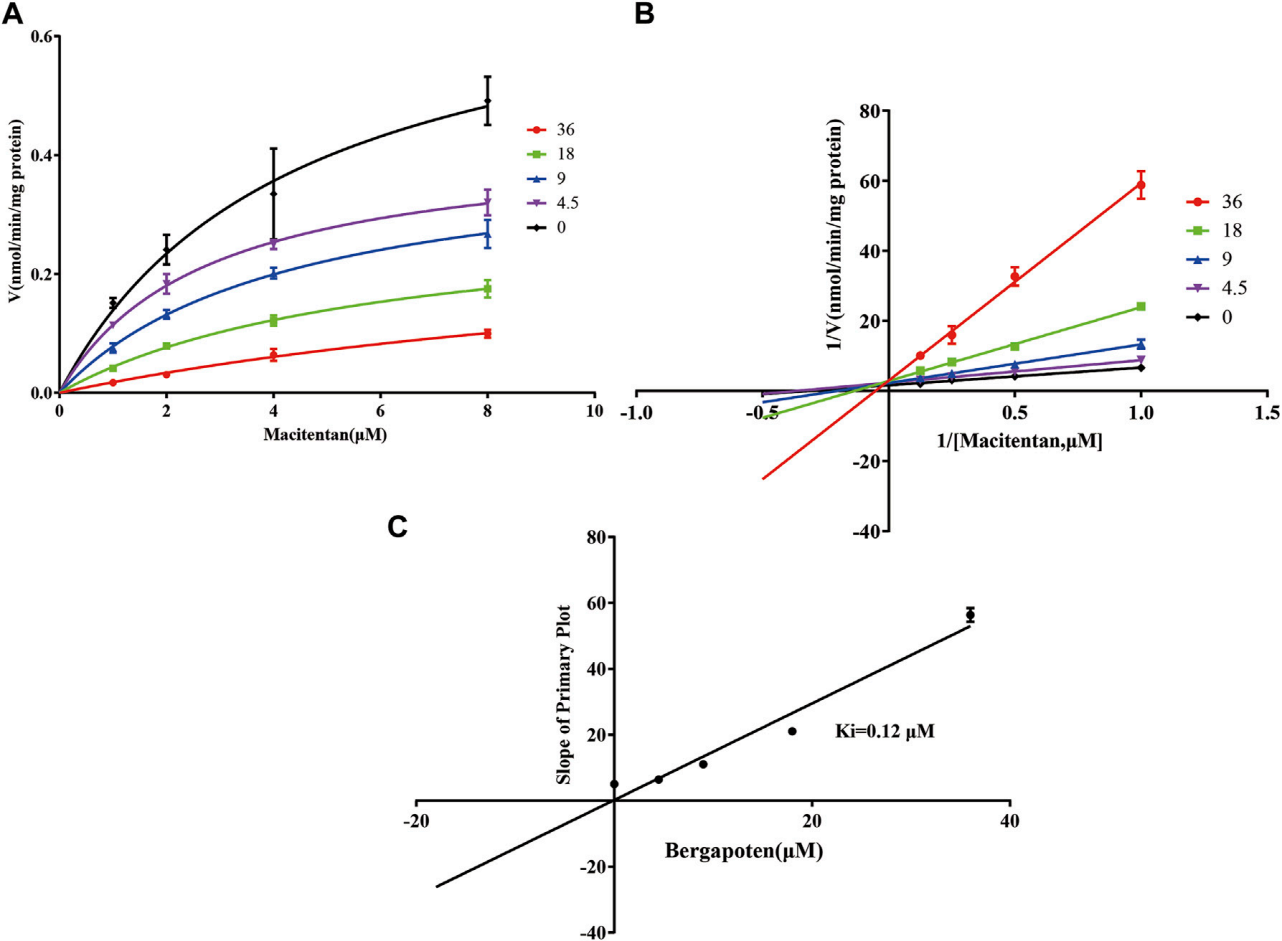
Michaelis-Menten model **(A)**, Lineweaver–Burk plots **(B)**, and slope of primary plot **(C)** for bergapten inhibition of macitentan in HLMs (*n* = 3, mean ± SD).

**FIGURE 6:**
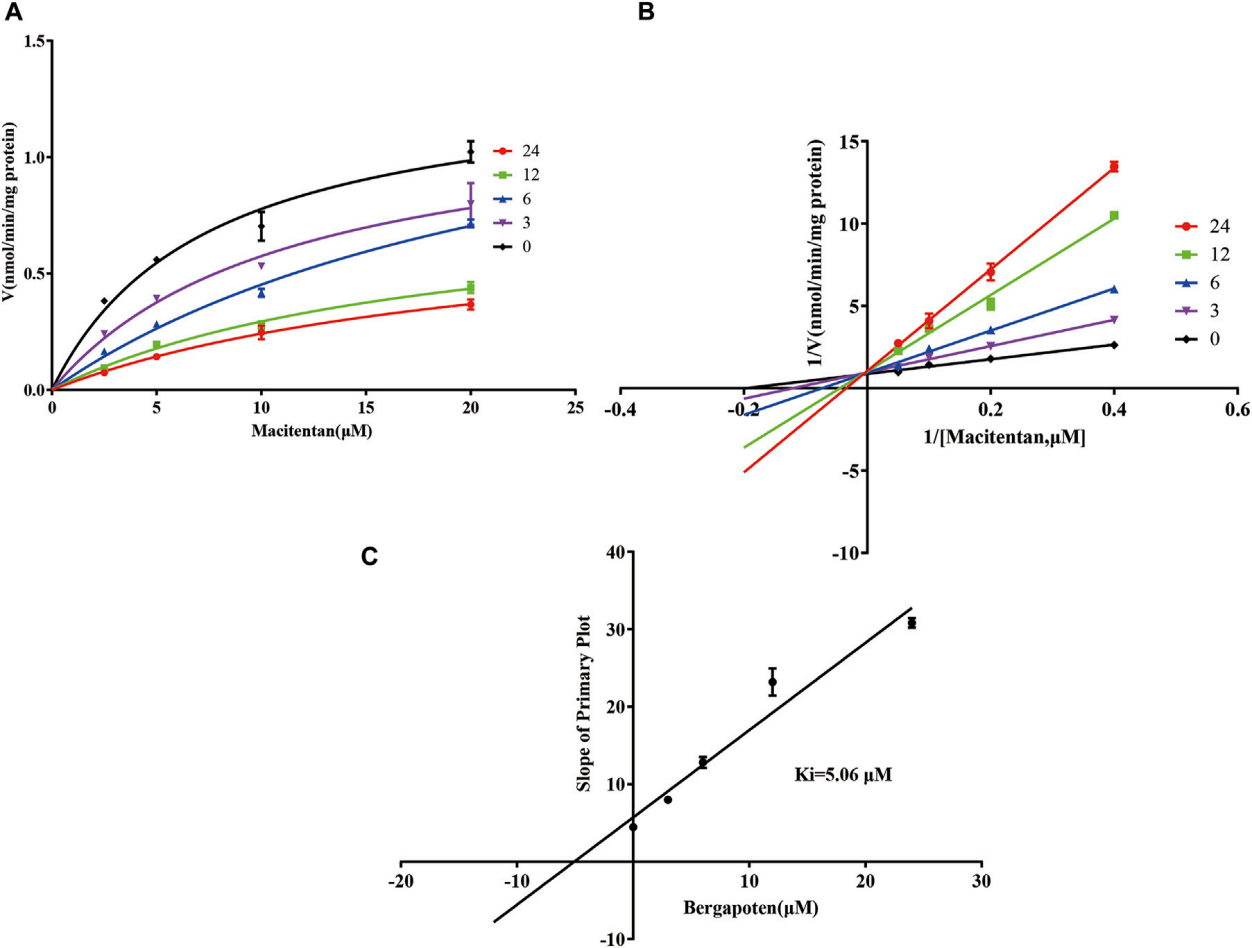
Michaelis-Menten model **(A)**, Lineweaver–Burk plots **(B)**, and slope of primary plot **(C)** for bergapten inhibition of macitentan in rCYP3A4 (*n* = 3, mean ± SD).

Firstly, we successfully established the methods for the quantification of macitentan and ACT-132577 (Figure 2). As shown in Figure 3, BP inhibited macitentan metabolism of with IC_50_ values of 3.84 μM in RLMs, 17.82 μM in HLMs, and 12.81 μM in rCYP3A4. The K_m_ values and V_max_ were 4.82 μM, 0.50 nmol/min/mg protein in RLMs, 3.59 μM, 0.94 nmol/min/mg protein in HLMs, and 13.14 μM, 2.32 nmol/min/mg protein in rCYP3A4, respectively. These outcomes indicated that BP moderately inhibited macitentan metabolism in RLMs, while weak inhibition was observed in HLMs and rCYP3A4. For further evaluation of the inhibition mechanisms of BP on macitentan, the Lineweaver–Burk plots are displayed in Figures 4–6. BP inhibited macitentan metabolism by competitive inhibition, with K_i_ values of 1.39, 0.12, and 5.06 μM in RLMs, HLMs, and rCYP3A4, respectively.

### 3.3 Evaluation of the influence of BP on macitentan metabolism and pharmacokinetics *in vivo*

**FIGURE 7:**
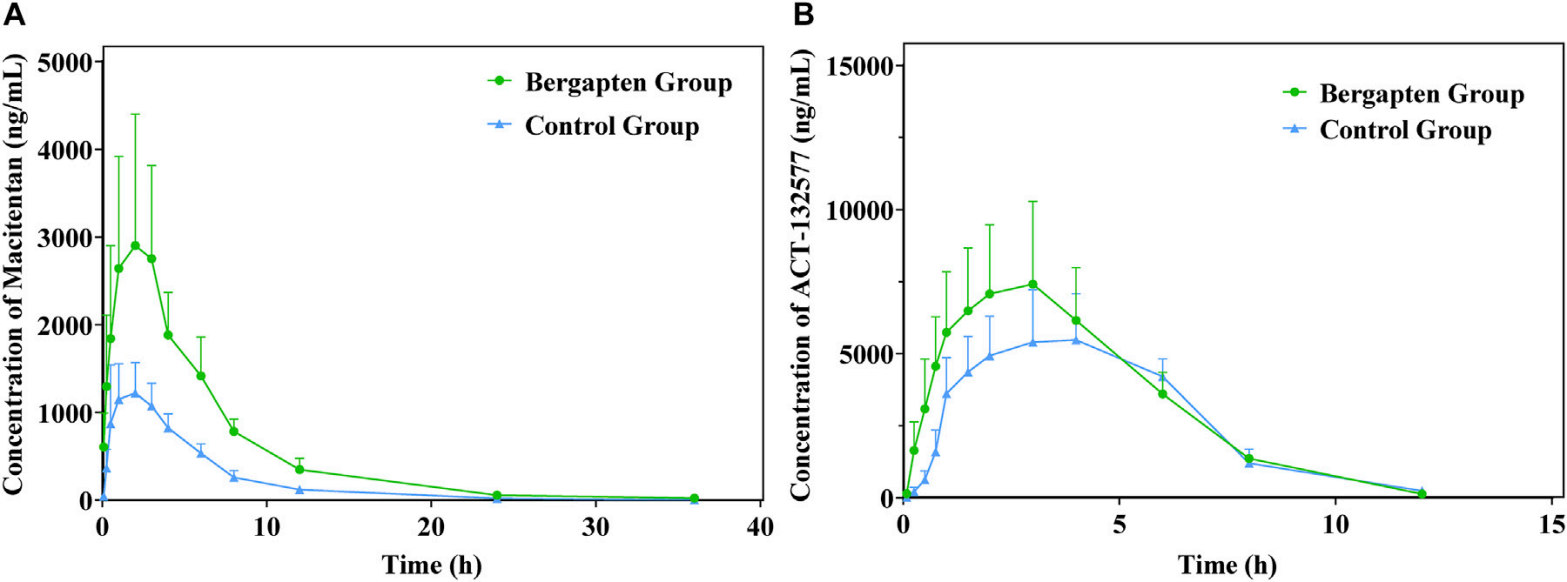
Mean plasma concentration-time profiles of macitentan **(A)** and ACT-132577 **(B)** (*n* = 6, mean ± SD).

The mean plasma concentration-time profiles of macitentan and its metabolite ACT-132577, with or without intragastric administration of BP in rats, are plotted in Figure 7. The other relevant pharmacokinetic parameters of macitentan and ACT-132577 are presented in Tables 3, 4. In comparison with the control group, macitentan exposure in the experimental group was enhanced by 1.57-fold AUC_(0-t)_ (*p* < 0.05), 1.58-fold AUC_(0-∞)_ (*p* < 0.05), and 1.24-fold C_max_ (*p* < 0.05), while the CL_z_/F significantly decreased by 0.59-fold. Although there were no significant differences in the pharmacokinetic parameters of ACT-132577 (*p* > 0.05), the MR (MR = AUC_ACT-132577_/AUC_Macitentan_) of the experimental group significantly decreased by 56.24% compared to that of the control group. The other pharmacokinetic parameters were comparable, with no significant differences (*p* > 0.05). These results confirmed that BP inhibited macitentan metabolism in rats.

**TABLE 3:** The primary pharmacokinetic parameters of macitentan in two groups of rats. (*n* = 6, mean ± SD).

Parameters	Control group	Experimental group
AUC_(0-t)_ (μg/L*h)	7,873.97 ± 1,384.98	20,263.67 ± 6,427.63*
AUC_(0-∞)_ (μg/L*h)	7,897.83 ± 1,369.87	20,378.31 ± 6,442.74*
MRT_(0-t)_ (h)	5.48 ± 0.96	5.91 ± 0.74
MRT_(0-∞)_ (h)	5.60 ± 1.08	6.12 ± 0.80
t_1/2z_ (h)	4.53 ± 0.90	5.07 ± 1.15
T_max_ (h)	1.92 ± 0.80	2.50 ± 0.55
CL_z_/F (L/h/kg)	2.60 ± 0.48	1.07 ± 0.36*
C_max_ (μg/L)	1,339.44 ± 518.24	2,999.69 ± 1,428.35*

Notes: *Significantly different from control, *p* < 0.05.

**TABLE 4:** The primary pharmacokinetic parameters of ACT-132577 in two groups of rats. (*n* = 6, mean ± SD).

Parameters	Control group	Experimental group
AUC_(0-t)_ (μg/L*h)	93,689.35 ± 9,770.34	107,185.45 ± 22,066.22
AUC_(0-∞)_ (μg/L*h)	95,982.33 ± 9,790.28	108,373.12 ± 21,125.06
MRT_(0-t)_ (h)	11.26 ± 1.40	10.34 ± 1.43
MRT_(0-∞)_ (h)	12.06 ± 1.81	10.78 ± 1.97
t_1/2z_ (h)	5.97 ± 1.09	5.18 ± 1.30
T_max_ (h)	7.33 ± 2.73	6.33 ± 1.51
CL_z_/F (L/h/kg)	0.21 ± 0.02	0.19 ± 0.04
C_max_ (μg/L)	5,695.69 ± 1,631.05	7,741.50 ± 2,615.93

Notes: *Significantly different from control, *p* < 0.05.

### 3.4 In silico analysis of the inhibitory mechanism of BP on macitentan

**FIGURE 8:**
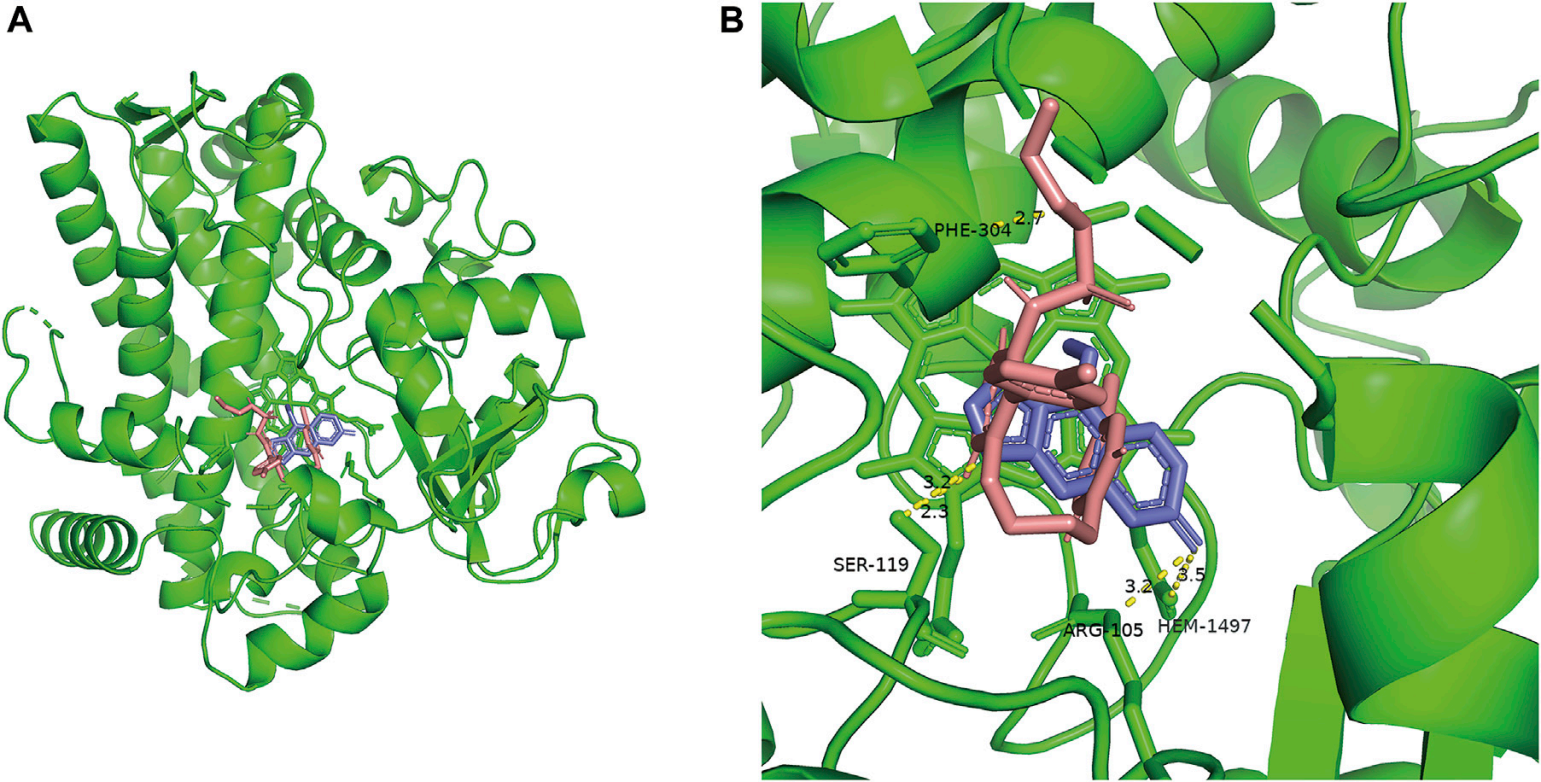
Overall view of the crystal structure of CYP3A4 and small ligands **(A)**. Detailed view of the crystal structure of CYP3A4 docked with bergapten (Blue) and macitentan (Pink) **(B)**.

As shown in Figure 8, the results of molecular docking illustrated that the inhibitor BP (Blue) and the substrate macitentan (Pink) can spontaneously bound to the active catalytic cavity of the target enzyme CYP3A4 with a binding energy of −6.3 and −7.4 kcal/mol, respectively. BP interacts with the active amino acid residues SER-119, ARG-105, HEM-1497 of enzyme through conventional hydrogen bonding at distances of approximately 3.2, 3.2, and 3.5 Å, respectively. Whereas macitentan forms two hydrogen bonds with the amino acid residues SER-119 and PHE-304 at distances of 2.3 and 2.7 Å. It was obvious that the binding areas of macitentan and BP were highly overlapped and the amino acid residue SER-119 was the shared binding site, which may be the main reason for the competitive inhibition of BP on the metabolism of macitentan through CYP3A4.

## 4 Discussion

PAH is a chronic disease characterized by a sustained increase in pulmonary vascular resistance, which causes inconvenience and heavy burden on people’s lives. Right ventricular failure and death occur if PAH is not treated (Galiè et al, 2013). Among the treatments for PAH, dual ETA/ETB receptor antagonists have excellent therapeutic efficacy. Macitentan is a novel dual endothelin receptor antagonist that was approved by the FDA in 2013 (Patel and McKeage, 2014). Macitentan displayed insurmountable antagonism, thus making an effective blocker of ET-1 (Gatfield et al, 2012). Given the dominant role of the CYP3A4 enzyme in macitentan metabolism, it is important to consider the potential DDIs that influence the efficacy and safety of PAH treatment. BP is a furanocoumarin that possesses a potent inhibitory effect on CYP3A4 and it is abundant in many Umbelliferae and Rutaceae plants which are widely included in many common Chinese medicine prescriptions (Ho et al, 2001). Our study is aimed to investigate the potential effects of BP on macitentan metabolism *in vitro* and *in vivo*. Besides, the *in silico* evaluation was also conducted to understand the interactions among inhibitors, substrates, and CYP3A4 enzymes.

In fact, liver slices, liver microsomes, hepatocyte and recombinant enzymes are the most common *in vitro* models for DDIs investigation in previous literature (Ekins et al, 2000; Zhou et al, 2011). In the present study, RLMs, HLMs, and rCYP3A4 were selected as for the *in vitro* experiments. Our results revealed BP to be a moderate inhibitor of macitentan with an IC_50_ value of 3.84 μM, while it acted as a weak inhibitor in HLMs and rCYP3A4, with IC_50_ values of 17.82 and 12.81 μM, respectively. Species differences between humans and rats may be responsible for this difference in inhibitory strength. To further ascertain the inhibitory effects of BP on macitentan metabolism, we investigated the inhibitory mechanisms in RLMs, HLMs, and rCYP3A4. The Lineweaver-Burk plots in Figure 4, 5, and 6 demonstrate that BP is a competitive inhibitor of macitentan in all incubation systems.

This *in vitro* study laid a foundation for the evaluation of the pharmacokinetics of macitentan with or without concurrent administration of BP in rats. According to Li’s report, the C_max_ value of bergapten in rat was around 447.72 ± 30.61 ng/mL (approximately 2.1 μM) when 3 mg/kg bergapten was administered (Li et al, 2014). To ensure getting adequate inhibitory concentration of bergapten *in vivo*, 20 mg/kg of bergapten were administered to rats in this study. As illustrated in Table 3, exposure to bergapten led to a significant decrease in CL_z_/F value but caused a substantial elevation of AUC_(0-t)_, AUC_(0-∞)_, and C_max_ of macitentan in experimental group. However, bergapten had no impacts on the t_1/2_ value but decreased the metabolite-parent AUC ratios (MR) in the experimental group. This result may be mainly due to the inhibitory effect of bergapten on the metabolic first-pass effects in the liver or small intestine (Choi et al, 2010; Park and Choi, 2019; Shi et al, 2019). It has been reported that macitentan was estimated to have an oral bioavailability of 74%, and it is mainly metabolized by CYP enzymes in the liver and intestine. Thus, macitentan may exhibit a weak first-pass effect after oral administration (Sidharta et al, 2015). It is plausible that the weakly gut and hepatic first-pass and hepatic systemic metabolism of macitentan may have been inhibited by BP, resulting in increased systemic exposure to macitentan. However, it seems unreasonable that the pharmacokinetic parameters of ACT-132577 in the experimental group were not significantly different from those of the control group. In view of this phenomenon, we traced this back to previous research. As a strong inhibitor of CYP3A4, ketoconazole was selected to assess its effect on macitentan pharmacokinetics. In the presence of ketoconazole, exposure to a single of dose macitentan increased approximately two-fold, whereas exposure to ACT-132577 only decreased by 26% (Atsmon et al, 2013). In another study, the presence of rifampin (a strong inducer of CYP3A4) resulted in a four-fold reduction in macitentan and no significant change in exposure to ACT-132577 (Bruderer et al, 2012b). Similarly, when macitentan was coadministered with St. John’s Wort (another well-known inducer of CYP3A), the concentrations of ACT-132577 remained unaltered, although exposure to macitentan was reduced to approximately half (Huppertz et al, 2018). It is evident that the pharmacokinetics of ACT-132577 was unaffected by BP in our study, which is in good agreement with previous studies. Although ACT-132577 also plays a role in the overall pharmacological effect of macitentan, it is approximately five-fold less potent than macitentan (Iglarz et al, 2008). As a result, the approximately 1.57-fold increase in exposure to macitentan in the presence of BP might cause problems with dose safety in clinical treatment. However, in light of the current animal experiment lacking clinical relevance and long-term safety data during the use of such a combination, more rigorous clinical trials should be conducted for further confirmation.

To better understand the interaction between BP and macitentan, a molecular docking study was performed to simulate the binding and possible trajectories of small ligands to the active cavity of the human CYP3A4 enzyme. Both the inhibitor (BP) and substrate (macitentan) could be docked into the active cavity of CYP3A4. More negative binding energy were formed between macitentan and CYP3A4 compared to BP, indicating that macitentan binds CYP3A4 more tightly. As the binding site of BP to the enzyme is highly overlapped to that of macitentan, BP may competitively inhibit macitentan, which provides a plausible explanation for the results of previous *in vitro* and *in vivo* studies.

## 5 Conclusion

In summary, BP competitively inhibits the metabolism of macitentan *in vitro* and alters its pharmacokinetic characteristics *in vivo*. *In silico* analysis simulates the interaction between two small molecules and the human CYP3A4 enzyme. Enhanced exposure to macitentan in the plasma as a result of BP coadministration may trigger pharmacodynamic alterations and side effects, implying that more attention should be paid to the combination of these two compounds. Owing to the limitations of the present study, the optimized and reasonable combination warrants further systematic clinical investigation.

## Data Availability

The original contributions presented in the study are included in the article/supplementary material, further inquiries can be directed to the corresponding authors.

## Abbreviations

AUC, area under the curve; BP, Bergapten; CL_z_/F, plasma clearance; C_max,_ maximal plasma concentration; CYP, cytochrome P450; DDI, drug-drug interactions; ET, endothelin; ETB, Endothelin (ET) type B receptors; ETA, Endothelin (ET) type A receptors; FDA, Food and Drug Administration; HLMs, human liver microsomes; MR, metabolite-parent ratio; PAH, pulmonary arterial hypertension; RLMs, Rat liver microsomes.
